# The Importance of Clinical History in the Etiological Diagnosis of Diffuse Alveolar Hemorrhage Associated With Improper Adrenaline Use

**DOI:** 10.7759/cureus.75727

**Published:** 2024-12-15

**Authors:** João Nuno Patrício, Ana Rafael, Filipe Conceição, José Manuel Pereira, José-Artur Paiva

**Affiliations:** 1 Department of Intensive Care Medicine, Hospital Beatriz Ângelo, Loures, PRT; 2 Department of Intensive Care Medicine, Centro Hospitalar De Trás-Os-Montes E Alto Douro, Vila Real, PRT; 3 Department of Intensive Care Medicine, Centro Hospitalar Universitário de São João, Porto, PRT; 4 Department of Medicine, Faculty of Medicine, University of Porto, Porto, PRT

**Keywords:** adrenaline, dyspnea, hemoptysis, pulmonary alveolar hemorrhage, respiratory failure

## Abstract

This case involves a 21-year-old male healthcare student with a medical history of HIV-1 infection for two years and anxiety disorder. He presented to the emergency department with hemoptysis and dyspnea of sudden onset. A thoracic CT scan revealed multiple bilateral nodular ground-glass opacities suggestive of diffuse alveolar hemorrhage (DAH). Due to hypoxemic respiratory failure, noninvasive ventilatory support was initiated. A bronchoalveolar lavage confirmed the presence of blood. He also showed an elevated troponin I, peaking at 2,915 ng/mL, with no electrocardiographic or echocardiographic abnormalities. Assuming an immune etiology, high-dose corticosteroids were initiated, with three days of methylprednisolone 1 g. However, the entire etiological study for DAH turned out negative. During a follow-up clinical interview, the patient admitted to having self-administered 1 mg of intravenous adrenaline shortly before the onset of symptoms. The patient showed a favorable evolution, with no recurrence of symptoms, allowing for oxygen therapy weaning. A follow-up chest CT on the ninth day of hospitalization showed no significant alterations. This case highlights the importance of a thorough history in determining the etiology of a potentially fatal disease, adding intravenous epinephrine as a potential trigger of hemoptysis due to exogenous agents.

## Introduction

Diffuse alveolar hemorrhage (DAH) is an uncommon but potentially life-threatening condition that requires prompt diagnosis and treatment. It is characterized by the accumulation of intra-alveolar erythrocytes. The most noticeable signs are the acute or subacute onset of cough, hemoptysis, and dyspnea, along with the appearance of radiographic pulmonary infiltrates and anemia, which may lead to hypoxemic respiratory failure [1].

Etiologically, it can be divided into infectious causes (affecting both immunocompromised and immunocompetent patients) and noninfectious causes, the latter being much more frequent [2]. Autoimmune diseases, coagulation disorders, and the use of drugs such as cocaine or other toxins stand out among these [3].

We present the case of a 21-year-old healthcare student who presented to the emergency department (ED) with sudden-onset dyspnea and hemoptysis. He was diagnosed with DAH following improper intravenous adrenaline administration, an association not previously described in the literature.

## Case presentation

We present the case of a 21-year-old male healthcare student. His medical history includes an HIV-1 infection diagnosed two years before, under antiretroviral therapy with an undetectable viral load, anxiety disorder on medication, and active smoking. He was referred to the ED for sudden-onset dyspnea with moderate hemoptysis. He denied other symptoms, including genitourinary, gastrointestinal, neurological, cutaneous, musculoskeletal, or chest pain. He also denied recent travels, new medications, or drug use. Upon initial physical examination, he presented with a blood pressure of 80/50 mmHg, heart rate of 70 bpm, respiratory rate of 24 breaths/minute, and oxygen saturation of 86% in room air. He had hemoptysis and cardiopulmonary auscultation with bilateral crackles and rhythmic heart sounds with no murmurs. There was no edema or other signs of deep vein thrombosis in the lower limbs. He was started on oxygen therapy with a Venturi mask (FiO_2_ of 31%). Arterial blood gas (ABG) analysis revealed a pH of 7.32, partial pressure of carbon dioxide (pCO2) of 43.9 mmHg, partial pressure of oxygen (pO2) of 68.6 mmHg, bicarbonate (HCO3) of 22.1 mmol/L, sulfur dioxide of 99.2%, and lactate of 2.9 mmol/L. Fluid therapy improved his blood pressure. His lab results (Table 1) showed acute kidney injury with creatinine 1.19 mg/dL and urea 40 mg/dL, neutrophilic leukocytosis with 15,111 leukocytes/L and 12,530 neutrophils/L, elevated high-sensitivity troponin I at 358 ng/L, and D-dimer at 3.62 µg/mL. Hemoglobin was 16.5 g/dL in the ED and 13.1 g/dL on intensive care unit (ICU) admission, and C-reactive protein was 3.1 mg/L. Urinalysis showed proteinuria (1 g/L) and albuminuria >150 mg/L, with no other abnormalities. The toxicology screening for drugs in urine was negative except for opiates, which were administered by the medical team.

**Table 1:** Lab values on ICU admission CRP: C-reactive protein; AST: aspartate transaminase; ALT: alanine transaminase; LDH: lactate dehydrogenase; GGT: gamma-glutamyl transferase; INR: international normalized ratio; ICU: intensive care unit

Test	Result	Reference range
Complete blood count		
Hemoglobin	13.1 g/dL	13.5-17.5 g/dL
Hematocrit	37%	41%-53%
Erythrocyte count	4.3 × 10^12^/L	4.3-5.9 × 10^12^/L
Platelet count	180 × 10^9^/L	150-400 × 10^9^/L
Mean corpuscular hemoglobin	30.5 pg	25-35 pg
Mean corpuscular hemoglobin concentration	38.2% Hb/cell	31%-36% Hb/cell
Leukocyte count	15.11 × 10^9^/L	4.5-11 × 10^9^/L
Neutrophils	12.53 × 10^9^/L	2.0-8.5 × 10^9^/L
Lymphocytes	1.53 × 10^9^/L	0.9-3.5 × 10^9^/L
Monocytes	0.83 × 10^9^/L	0.2-1.0 × 10^9^/L
Eosinophils	0.14 × 10^9^/L	0.0-0.6 × 10^9^/L
Biochemistry		
Glucose	128 mg/dL	<140 mg/dL
Creatinine	1.19 mg/dL	0.6-1.2 mg/dL
Urea	40 mg/dL	16.6-48.5 mg/dL
High-sensitivity troponin I	358 ng/L	<40 ng/L
D-dimer	3.62 µg/mL	<0.5 µg/mL
CRP	3.1 mg/L	<5.0 mg/L
Liver enzymes		
AST	27 U/L	10-40 U/L
ALT	16 U/L	12-38 U/L
LDH	230 U/L	45-200 U/L
Alkaline phosphatase	54 U/L	25-100 U/L
GGT	16 U/L	<60 U/L
Coagulation study		
Prothrombin time	12 seconds	9.5-12.5 seconds
Activated partial thromboplastin time	26.9 seconds	25-40 seconds
INR	0.91	0.8-1.2
Electrolytes		
Sodium	138 mEq/L	136-146 mEq/L
Potassium	4.12 mEq/L	3.5-5.0 mEq/L
Chloride	106 mEq/L	95-105 mEq/L
Total calcium	4.4 mEq/L	2.1-2.6 mEq/L
Phosphorus	2.5 mg/dL	3.0-4.5 mg/dL
Magnesium	1.44 mg/dL	1.5-2.0 mg/dL
Urinalysis		
Appearance	Clear	-
pH	5.5	5-8
Specific gravity	1.040	1.002-1.030
Proteins	1 g/L	<0.15 g/L
Ketones	Negative	-
Glucose	Negative	-
Nitrites	Negative	-
Leukocyte count	24.3/µL	<15/µL
Erythrocyte count	Negative	-
Benzodiazepines	Negative	-
Barbiturates	Negative	-
Cannabinoids	Negative	-
Cocaine	Negative	-
Amphetamines	Negative	-
Opiates	Positive	-

**Figure 1:**
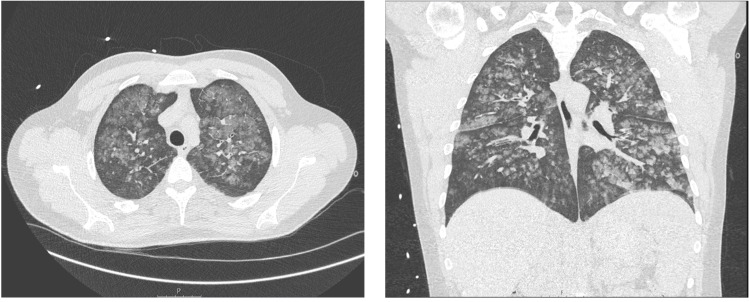
Thoracic CT scan on admission. Left: axial view. Right: coronal view

A thoracic CT scan (Figure 1) revealed multiple nodules with ground-glass opacity scattered throughout the bilateral lung parenchyma, predominantly central, suggestive of indeterminate alveolar hemorrhage, with the possibility of superimposed or associated atypical pneumonia. There were no signs of pulmonary embolism. Other investigations for diagnostic workup did not reveal any relevant abnormality (Table 2).

**Table 2:** Diagnostic workup dsDNA: double-stranded DNA; ANCA: antineutrophil cytoplasmic antibodies; CCP: cyclic citrullinated peptide; ACE: angiotensin-converting enzyme; RSV: respiratory syncytial virus; BAL: bronchoalveolar lavage; IGRA: interferon gamma release assay

Test	Result	Reference range
Rheumatoid factor	Negative	-
ANA	Negative	-
Anti-dsDNA antibodies	Negative	-
Antihistone antibodies	Negative	-
ANCA	Negative	-
Antiglomerular basement membrane antibodies	Negative	-
Anticardiolipin antibodies	Negative	-
Anticentromere antibodies	Negative	-
Antigliadin antibodies	Negative	-
Anti-CCP antibodies	Negative	-
Antitransglutaminase antibodies	Negative	-
ACE	48 U/L	20-70 U/L
Complement study (C3c, C4, and C5)	No alterations	-
Peripheral blood smear	No alterations	-
Protein electrophoresis	No alterations	-
Serum immunoglobulins (G, A, and M)	No alterations	-
Light chains (kappa and lambda)	No alterations	-
Haptoglobin	43 mg/dL	50-320 mg/dL
Schistocyte screen	Negative	-
Serum DNA test for leptospira	Negative	-
Respiratory virus panel (SARS-CoV-2, RSV, influenza A and B)	Negative	-
Legionella antigenuria	Negative	-
Streptococcus pneumoniae antigenuria	Negative	-
Blood cultures	Negative	-
Urine culture	Negative	-
Bronchial wash bacteriological exam	Negative	-
Bronchial wash mycobacteriological exam	Negative	-
BAL bacteriological exam	Negative	-
BAL mycobacteriological exam	Negative	-
BAL immunophenotyping	No alterations	-
Mycobacterium tuberculosis DNA in bronchial secretions	Negative	-
IGRA	Negative	-
Erythrocyte sedimentation rate	49 mm/first hour	<15 mm/first hour

In the ED, he experienced several episodes of hemoptysis and type 2 respiratory failure (ABG: pH of 7.29, pCO_2_ of 50 mmHg, pO_2_ of 86 mmHg, HCO_3_ of 23.5 mmol/L), necessitating noninvasive ventilation with bilevel positive airway pressure mode and subsequent transfer to the ICU. He had a rising troponin curve, peaking at 2,915 ng/L, without associated chest pain and any electrocardiographic or echocardiographic abnormalities. After discussion with the Pulmonology Team, DAH of autoimmune cause with the lung-kidney syndrome was assumed, leading to an immunological study and initiation of high-dose corticosteroid therapy with 1 g of methylprednisolone for three days, later changed to 1 mg/kg/day for another six days. Fiberoptic bronchoscopy with bronchoalveolar lavage (BAL) revealed macroscopically hemorrhagic characteristics and the presence of numerous intact erythrocytes. Hemosiderin staining in the cytoplasm of macrophages using Prussian blue staining revealed a Golde score of 19, indicating normal results.

**Figure 2:**
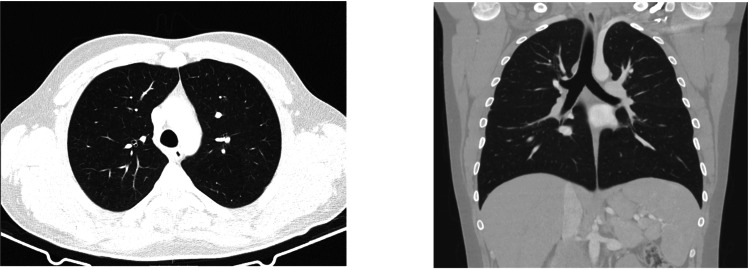
Thoracic CT scan on the ninth day of hospitalization. Left: axial view. Right: coronal view

The patient did not experience further hemoptysis after the second day of hospitalization, with overall clinical improvement allowing weaning from ventilatory support to high-flow oxygen therapy and subsequent titration to conventional oxygen therapy, which was suspended on the third day. He was transferred to the Internal Medicine ward on the third day. A follow-up thoracic CT scan on the ninth day (Figure 2) showed almost complete resolution of the ground-glass opacities, with only a 3-mm ground-glass nodule in the anterior segment of the left upper lobe.

During a subsequent clinical interview, the patient revealed that he had self-administered 1 mg of intravenous adrenaline through peripheral venous access to experiment with the drug's effects. He was evaluated by the Psychiatry Team in this context, expressed regret for the incident, and was found to have no psychiatric disorders, including suicidal ideation. The patient was discharged on the 12th day of hospitalization, asymptomatic, and eupneic in room air, with an oxygen saturation of 99%. Cardiopulmonary auscultation was unremarkable, and all previously abnormal lab abnormalities had resolved.

## Discussion

DAH is a rare but potentially serious condition. This patient exhibited symptoms consistent with this entity, which often presents with nonspecific symptoms such as hemoptysis, chest pain, dyspnea, and cough; radiological signs of bilateral diffuse infiltrates; and hypoxemic respiratory failure [2,3]. The most common causes in young individuals are autoimmune diseases, such as vasculitis with capillaritis or pulmonary vasculitis, as well as drug use, particularly cocaine [1,3,4]. However, the patient's immune workup was negative, and toxicology screening for drugs in urine was also negative. Infectious causes should also be considered, although infection is rarely associated with DAH [2]. It should be noted that the patient was HIV-positive, which, if there was poor adherence to antiretroviral therapy, could increase the risk of infections from atypical bacteria, fungi, or viruses. However, all microbiological samples, including BAL, were negative. Coagulation disorders were also ruled out by the tests performed. In addition to the clinical presentation, the diagnosis of DAH was supported by the presence of diffuse infiltrates on chest X-ray and CT scan and by bloody BAL. Another indicator of this condition was the patient's drop in hemoglobin from 16.5 g/dL on admission to a minimum of 11.6 g/dL within the first 48 hours of hospitalization. The timing of the exam might explain the absence of hemosiderin-laden macrophages (HLMs) in BAL microscopic examination. Fiberoptic bronchoscopy was performed in the first 48 hours of the onset of symptoms, while HLM started to appear on the third day, with a peak on the seventh day [5]. Considering the history of intravenous adrenaline self-administration, the authors hypothesize that pulmonary and bronchial vessel vasoconstriction and microcirculation disruption, caused by the drug and resulting in acute hypertension, are the most likely causes of alveolar hemorrhage in this patient. The increased pressure likely disrupted the alveolar-capillary membrane, allowing red blood cells to accumulate in the alveolar spaces. Additionally, drug-induced vasoconstriction may have led to anoxic cell damage. Moreover, the β1 effect of the drug could have increased heart rate and myocardial contractility, potentially causing some degree of secondary myocardial injury, explaining the transient elevation in troponin levels without associated electrocardiographic or echocardiographic abnormalities, which later normalized. Cases of acute myocardial infarction following adrenaline administration have been described in the literature [6-8]. The favorable evolution over a short period, albeit intravenous corticosteroid therapy, suggests a secondary phenomenon resulting from an episodic insult without ongoing impact. The patient's clinical and radiological progression supports the hypothesis of a single, self-limited episode, with rapid spontaneous resolution. This type of evolution is also similar to what has been reported in other drug use cases [4]. This, in conjunction with a negative immunological panel, makes the hypothesis of a primary vasculitic phenomenon less likely.

## Conclusions

DAH is a rare, potentially fatal condition that requires rapid diagnosis and treatment. Its etiology is diverse and often uncertain. Immune causes, infections, and substance use must be investigated. Although there have been reports of DAH associated with various toxins, the authors found no publication linking DAH to therapeutic or nontherapeutic intravenous adrenaline administration. Therefore, reporting this case is important, as it underscores the crucial role of clinical history in managing this condition.
